# The foot-health of adult diabetics in regional Australia: baseline findings from an epidemiological study

**DOI:** 10.1186/1757-1146-8-S2-O32

**Published:** 2015-09-22

**Authors:** Byron Perrin, Penny Allen, Isabelle Skinner, Marcus Gardner, Andrew Chappell, Bronwyn Phillips, Claire Massey, Timothy Skinner

**Affiliations:** 1 La Trobe Rural Health School, La Trobe University, Bendigo, 3550, Australia; 2 Rural Clinical School, University of Tasmania, Launceston, 7250, Australia; 3 School of Psychological and Clinical Sciences, Charles Darwin University, Darwin, 0800, Australia; 4 Outpatient Rehabilitation Services, Bendigo Health, Bendigo, 3550, Australia; 5 Tasmanian Health Organisation- North-West, Devonport, 7310, Australia; 6 Bendigo Community Health Services, Bendigo, 3550, Australia; 7 Tasmanian Health Organisation- North, Launceston, 7250, Australia

**Keywords:** Rural Health, Epidemiology, Diabetes, Public Health

## Background

There is limited epidemiological research that reports on the foot-health of people with diabetes within Australian regional settings. This study reports on the baseline characteristics of a large prospective cohort study. The objective of this baseline analysis is to explore the relationship between demographic, socio-economic and diabetes-related variables with diabetes-related foot morbidity in people residing in regional and rural Australia.

## Methods

Adults with diabetes were recruited from predominately community-based, publicly-funded podiatric services in regional Victoria and Tasmania. The primary variable of interest was the University of Texas diabetic foot risk classification designated to each participant at baseline. Other variables of interest were state of residence, socio-economic disadvantage, rurality, age, sex, diabetes type and duration, knowledge of diabetes and smoking status. A multivariate generalised ordered logit model was built to identify independent associations with foot morbidity.

## Results

Nine hundred and three patients with diabetes were recruited. Mean age was 67 years (SD 12.6), with a majority being male (56.8%) and having diabetes for longer than 10 years (56.0%). The proportion of participants recruited was equivalent in each state. Socio-economic status was low with 59.0% of participants residing in the third most socio-economically deprived postcodes. Half of the participants indicated poor diabetes knowledge. A majority of the sample had neuropathy or worse foot morbidity (n=554, 61.4%). Worse foot morbidity was associated with male sex (OR 2.49, 95%CI 1.83-3.38) and duration of diabetes >20 years (OR 3.42, 95%CI 2.42-4.83), with Tasmanian residents having triple the odds of worse foot morbidity (OR 3.21, 95%CI 2.23-4.83).

## Conclusions

It is important to recognise that Australian regionally-based public podiatric services are managing a high proportion of patients at significant risk of future diabetes-related foot morbidity, especially in Tasmania. These patients are socially disadvantaged and have disappointingly low levels of knowledge about their diabetes. Regional public podiatric services are predominately funded by community-based funding schemes that may not support the multi-disciplinary health care that these patients need as recommended by national guidelines. There is a potential disparity between current funding models for public regional podiatry care and the level of diabetes-related foot morbidity the services are managing.

